# Extracellular Vesicle Proteins Associated with Systemic Vascular Events Correlate with Heart Failure: An Observational Study in a Dyspnoea Cohort

**DOI:** 10.1371/journal.pone.0148073

**Published:** 2016-01-28

**Authors:** Ya-Nan Zhang, Flora Vernooij, Irwani Ibrahim, Shirley Ooi, Crystel M. Gijsberts, Arjan H. Schoneveld, Kuan Win Sen, Hester M. den Ruijter, Leo Timmers, Arthur Mark Richards, Chun Tzen Jong, Ibrahim Mazlan, Jiong-Wei Wang, Carolyn S. P. Lam, Dominique P. V. de Kleijn

**Affiliations:** 1 Department of Surgery, Yong Loo Lin School of Medicine, National University of Singapore, Singapore, Singapore; 2 Cardiovascular Research Institute, National University Heart Centre Singapore; National University Health System Singapore, Singapore, Singapore; 3 Department of Emergency Medicine, National University Health System, Singapore, Singapore; 4 Laboratory of Experimental Cardiology, University Medical Center Utrecht, Utrecht, the Netherlands; 5 Interuniversity Cardiology Institute of the Netherlands, Utrecht, the Netherlands; 6 Christchurch Heart Institute, University of Otago, Christchurch, New Zealand; 7 National Heart Centre Singapore, Singapore General Hospital, Duke-National University of Singapore, Singapore, Singapore; Scuola Superiore Sant'Anna, ITALY

## Abstract

**Background:**

SerpinF2, SerpinG1, CystatinC and CD14 are involved in inflammatory processes and plasma extracellular vesicle (EV) -levels of these proteins have been reported to be associated with systemic vascular events. Evidence is accumulating that inflammatory processes may play a pivotal role both in systemic vascular events and in heart failure. Therefore, we studied the association between plasma extracellular vesicle SerpinF2-, SerpinG1-, CystatinC and CD14-levels and the occurrence of acute heart failure in patients.

**Methods and Result:**

Extracellular vesicle protein levels of SerpinG1, SerpinF2, CystatinC and CD14 were measured in an observational study of 404 subjects presenting with dysponea at the emergency department (4B-cohort). Plasma extracellular vesicles were precipitated in a total extracellular vesicles (TEX)-fraction and in separate LDL- and HDL-subfractions. Extracellular vesicle protein levels were measured with a quantitative immune assay in all 3 precipitates. Out of 404 subjects, 141 (35%) were diagnosed with acutely decompensated heart failure. After correction for confounders (including comorbidities and medications), levels of CD14 in the HDL-fraction (OR 1.53, p = 0.01), SerpinF2 in the TEX-and LDL-fraction (ORs respectively 0.71 and 0.65, p<0.05) and SerpinG1 in the TEX-fraction (OR 1.55, p = 0.004) were statistically significantly related to heart failure. Furthermore, extracellular vesicle CD14- and SerpinF2-levels were significantly higher in heart failure patients with preserved ejection fraction than in those with reduced ejection fraction.

**Conclusion:**

Extracellular vesicle levels of CD14, SerpinG1 and SerpinF2 are associated with the occurrence of heart failure in subjects suspected for acute heart failure, suggesting common underlying pathophysiological mechanisms for heart failure and vascular events.

## Introduction

### Background

Plasma extracellular vesicles (EVs) are bilayer membrane vesicles including exosomes, microvesicles and microparticles [1]. They play an important role in intercellular communication and contain proteins, miRNA and mRNA from the cell of origin, reflecting its physiological or pathological status [1]. Furthermore, distinct bilayer membrane plasma vesicles co-fractionate with monolayer Low-Density Lipid particles (LDL), while other bilayer membrane vesicles co-fractionate with High-Density Lipid particles (HDL) [2]. This allows separation of distinct plasma EV-sub-fractions via sequential LDL and HDL isolation to identify subpopulations of EV-proteins each with its own protein content and associated pathophysiological pathways.

Plasma EVs are increasingly under investigation as vehicles for proteins that play a role in cardiovascular disease [3]. Plasma EV-counts are reported to be markers of endothelial dysfunction associated with future cardiovascular events [4, 5] and have also been associated with cardiometabolic risk in the Framingham cohort [6]. Furthermore, increased numbers of plasma EVs have also been associated with heart failure (HF). CD144+ endothelial-derived vesicle counts increase with NYHA class [7] and CD31+/Annexin V+ vesicle counts are associated with HF-related death and recurrent hospitalization [8].

The content of plasma EVs, however, has not yet been explored extensively. Using a proteomics approach, we recently identified the extracellular vesicle proteins (EV-proteins) CystatinC, SerpinF2, SerpinG1 and CD14 as markers of vascular events and mortality in patients with clinically manifest vascular disease [9]. CystatinC is associated with decreased renal function and Acute Coronary Syndrome [10, 11] and with an increased risk of systemic vascular events in symptomatic patients with cardiovascular disease [12]. SerpinG1, also known as C1-inhibitor, is an inhibitor of the complement system and several fibrinolytic and coagulation system proteases and is also involved in vascular permeability [13]. SerpinF2 (alpha 2 antiplasmin) reduces fibrinolysis via inhibition of plasmin but also influences inflammation and tissue remodeling [14]. Similarly, CD14, as co-receptor of Toll-like receptor 4, is involved in inflammation, while monocyte derived EVs, marked by CD14, are pro-coagulant [15]. Thus, these 4 EVs markers for systemic vascular events have been described in thrombotic and inflammatory processes.

Vascular inflammation is also increasingly recognized to contribute to heart failure (HF). Heart failure can be classified based on the ejection fraction (EF) as HF with reduced EF (HFREF) or HF with preserved ejection fraction (HFPEF).

In patients with HFREF, coronary artery disease is the major risk factor with underlying systemic atherosclerosis and comorbidities [16]. Systemic plasma levels of pro-inflammatory cytokines like TNF alpha and IL6 are higher in HF patients [17]. Furthermore, production of TNF alpha and IL6 is regulated by the Toll-like receptors of the innate immune system. In animal HFREF models, Toll-like receptor-deficient blood cells and not the heart determine post-MI loss of ejection fraction independent of infarct size [18, 19]. This demonstrates that systemic inflammation plays a causal role in post MI adverse LV remodeling, a precursor to HFREF [20].

Likewise for HFPEF, a systemic pro-inflammatory state associated with various comorbidities is proposed [21, 22]. Paulus and Tschope suggest that in HFPEF, comorbidities including hypertension, obesity and diabetes, induce a systemic pro-inflammatory state leading to coronary microvascular inflammation which drives cardiac remodeling and diastolic dysfunction [22].

Systemic vascular disease, inflammation and plasma EV-counts have been associated with HF. However, EV-content and, more specifically, EV systemic vascular event-markers have not been investigated in relation to the occurrence of heart failure.

### Objectives

In this study, we hypothesized that the recently identified 4 plasma EV proteins associated with systemic vascular events are also associated with the occurrence of HF. These plasma EV-proteins have been measured in the total vesicle-population and in distinct vesicle sub-populations.

Plasma sub-fraction levels of EV CystatinC, SerpinF2, SerpinG1 and CD14 were measured in a cohort of patients presenting with breathlessness at the emergency department (4B-cohort). We investigated the relationship between these EV proteins and HF, and secondarily between these EV proteins and HFREF or HFPEF.

## Material and Methods

### Ethics statement

The Domain Specific Review Board of National Health Group of Singapore (Reference number: 2012/0090) approved the study and written informed consent was obtained from all patients. This study conforms to the Declaration of Helsinki.

### Study design

As an observational, cross-sectional study, the operators of the experiments before data analysis were blinded of the grouping. The Extracellular Vesicles (EVs) were precipitated wholly or fractionatedly from each available plasma sample in the cohort with a uniform protocol. After the precipitation, EVs were lysed in order to release all content, the empirically selected proteins were measured quantitatively with commercial immunoassay. The results were analyzed between HF cases and controls, and subsequently subgroups, heart failure with preserved (HFPEF) or reduced ejection fraction (HFREF). The available confounders were used for further correction and adjustment.

### Patients

The study population consisted of patients included in the prospective, single-center 4B (Biomarkers Beyond BNP in Breathless patients)-cohort study. Between 2010 and 2013, 4B included patients over 21 years of age who attended the emergency department (ED) of the National University Hospital Singapore during daytime with the primary complaint of shortness of breath. Patients who required immediate intensive care or intubation or patients who were unable or unwilling to give consent were not included. Furthermore, patients who were on dialysis or had obvious trauma related shortness of breath (e.g. crush injury or penetrating wounds) were excluded.

Blood was sampled on admission and a standardized information set on patient history, symptoms and clinical findings was collected. All blood samples were collected in EDTA tubes and spun at 4,000g for 10 min at 4°C to obtain plasma. Plasma samples were stored at -80°C.

The HF group consisted of patients with acutely decompensated heart failure (ADHF) at the index acute presentation. Patients with a history of established HF and recent onset breathlessness that was not diagnosed as ADHF were excluded from the control group, for it was unclear to what extend HF contributed to the current complaints.

### Diagnoses

Diagnoses of heart failure were adjudicated according to the criteria of the guidelines issued by the European Society of Cardiology [23]. Subjects were diagnosed as HF patients when they showed HF symptoms and signs of cardiac dysfunction and/ or elevated N-terminal pro B-type natriuretic peptide (NT-proBNP). The cutoffs of NT-proBNP were 450 pg/mL for patients under 50-year-old, 900 pg/mL for patients between 50- and 75-year old, and 1800 pg/mL for patients over 75-year-old, respectively [23]. For those with a doubtful HF diagnosis, the response to treatment was assessed. Diagnoses were independently assessed by two reviewers (MR, cardiologist and II, emergency physician) based on all available clinical information. They were blinded to each other’s diagnosis, to NT-proBNP levels and to the EV-protein levels.

Furthermore, the New York Heart Association classification was assessed by the two reviewers.

Heart failure with preserved (HFPEF) or reduced ejection fraction (HFREF) was adjudicated based on recent echocardiography data. HFREF was defined as presence of the clinical syndrome of HF in the presence of a left ventricular ejection fraction equal to or less than 50% whereas patients with HFPEF had an ejection fraction higher than 50%.

As some overlap between diagnoses was possible, a subgroup analysis was performed. Control-patients with a NT-proBNP < 400 pg/ml were selected to exclude concurrent mild heart failure. Furthermore, since the EV-proteins that were measured, were associated with vascular events, we selected a subgroup of HF-patients without signs of myocardial ischemia. Patients with a low high-sensitive-TroponinT (hsTnT) and without ECG-manifestations of myocardial ischemia were included in this subgroup. An hsTnT-cutoff of 20 ng/L was chosen because it has been shown that patients with chronic stable heart failure have increased hsTnT [24].

Anemia was defined according to World Health Organization guideline as a hemoglobin level less than 120 g/L in women and less than 130 g/L in men [25].

The glomerular filtration rate (GFR) at admission to the emergency department was available for 122 patients. Creatinine-levels were available for 373 patients. Using the Cockcroft-Gault formula, creatinine clearance was estimated in these patients as follows: ((140-age (in yr.))*body weight (in kg) *constant)/serum creatinine (in μmol/L), with constant = 1.23 for men and 1.04 for women. Subsequently a reduced GFR was defined as a GFR < 60 ml/min/1.73m^2^, or, if GFR was missing but the creatinine clearance could be estimated, a creatinine clearance <60 ml/min.

### Isolation of Extracellular Vesicle Plasma subfractions

As previously described [26], Dextran Sulphate (DS) and Manganese (II) chloride (MnCl_2_) solution was used to precipitate LDL and the HDL plasma fractions. Briefly, a stock of DS and MnCl_2_ were prepared as 6.5% and 2M solutions respectively.

For the Total Extracellular vesicle (TEX) fraction, Xtractt buffer (Cavadis BV; 1:4) was added to 125μl plasma and mixed. The mixture was incubated at 4°C for 30 min and subsequently centrifuged at 4,800g at 4°C for 10 min. This pellet was dissolved in 125μL Roche lysis buffer and used in the quantitative magnetic bead assays as the TEX fraction.

For precipitation of the LDL fraction, 1μL DS stock and 3.1μL MnCl_2_ stock were added into 125 μL plasma and mixed. The mixed sample was centrifuged for 10 min at 4,800g at 4°C precipitating the LDL fraction pellet. This pellet was dissolved in 125μL lysis buffer (Roche # 04719956001) and used in the quantitative magnetic bead assays as the LDL fraction.

For the HDL fraction, 60 μL of supernatant above the LDL pellet was transferred to a new tube topped up with 65 μL Phosphate-Buffered-Saline (PBS) and mixed. Next, 12.5μL DS stock and 12.5μL MnCl_2_ stock were added into the 125 μl diluted supernatant and mixed. Subsequently, the sample was incubated for 2 h at 4°C and the sample was centrifuged for 10 min at 4,800g at 4°C to collect the HDL fraction pellet. This pellet was dissolved in 125 μL Roche lysis buffer and used in the quantitative magnetic bead assays as the HDL fraction.

The presence of the EVs was verified by the expression of CD9 (S3 Fig) and the morphology under electron microscopy (S2 Fig) [27].

### Quantitative Magnetic Bead Assays

Quantitative analysis of the selected proteins was conducted as previously described [21]. Briefly, MagPlex-C Microspheres (Luminex # MC100xx-01) were conjugated with the selected antibodies to synthesize the bead-capture antibody complex. Samples were incubated with the bead-capture antibody complex and subsequently with the biotinylated antibodies to detect the captured protein. Streptavidin-Phycoerythrin (SA-PE, BD bioscience # 554061) was added to quantify the concentration of captured protein. Standard curves were correlated with PE signal and dilution of homologous recombinant proteins. For readout and data processing system the Bio-Plex^®^ 200 Systems (Bio-Rad # 171–000201) was used. The antibodies and recombinant proteins used in Bioplex detection of CystatinC were anti-Human CystatinC (R&D Systems # MAB11962), biotinylated anti-Human CystatinC (R&D Systems # BAM11961) and recombinant Human CystatinC (R&D Systems #1196-PI). For CD14, anti-Human CD14 (R&D Systems #MAB3832), biotinylated anti-Human CD14 (R&D Systems #BAF383) and recombinant Human CD14 (R&D Systems #383-CD) were used. For SerpinF2, anti-Human SerpinF2 (R&D Systems #MAB1470), biotinylated anti-Human SerpinF2 (R&D Systems #BAF1470) and recombinant Human SerpinF2 (R&D Systems #1470-PI-010) were used and for SerpinG1, anti-Human SerpinG1 (R&D Systems #MAB2488), biotinylated anti-Human SerpinG1 (R&D Systems #BAF2488) and recombinant Human SerpinG1 (R&D Systems #2488-PI) were used.

### Statistical Analyses

Differences in baseline-characteristics were analyzed using Chi-square test for categorical variables, T-tests for normally distributed continuous variables and Mann-Whitney-U-tests for continuous variables that were not normally distributed.

The outcomes include the protein level of SerpinG1, SerpinF2, CystatinC and CD14 in the Total Extracellular vesicle (TEX) fraction, LDL fraction and HDL fraction.

To calculate the odds ratio and enable the direct comparison between different proteins and fractions, EV-protein levels were converted into standard deviation units, or the z-score, by using the observed value minus the mean value, divided by the standard deviation.

Undetectably low SerpinF2-levels were imputed in 20 patients by inserting half of the lowest detectable value.

To investigate the relationship between the protein-level and HF, z-scores of the protein-levels were introduced univariably into a logistic regression model with the outcome HF. Subsequently, 2-way ANOVA was used to investigate whether potential confounders also influenced the level of the proteins. Potential confounders were age, gender, medications and comorbid conditions. Log transformed values of the protein-levels were used to reduce the effect of skewness in the distribution of the protein-levels. Both the potential confounders and HF were entered into the 2-way ANOVA and only those confounders with p-value<0.05 were selected for multivariable correction. Next, a multivariable logistic regression model was made using 2-step logistic regression for each protein in each fraction. The proteins were entered in the first block and the confounders were selected by backward selection in the second block. In this model, interactions with history of myocardial ischemia, age, gender, hypertension, diabetes and chronic renal failure were tested. For history of myocardial ischemia a significant interaction was found, hence stratified analysis was performed.

All statistics was achieved by SPSS^®^ (IBM^®^, Version 22.0.0.0) except that the correlation plots were built by Rstudio [28] and R software [29] for statistical computing version 3.1.2.

## Results

### Baseline characteristics

Frozen plasma samples were available from 421 patients out of the total 617 patients of the 4B-cohort (Flowchart: S1 Fig). 17 patients with a history of congestive heart failure but no diagnosis of HF at the time of inclusion were excluded. Table 1 shows the baseline characteristics of the remaining 404 patients. Out of the 404 patients, 141 (35%) were adjudicated as having acute decompensated heart failure (ADHF). Patients in the remainder of the breathless population acted as the control group and were most often diagnosed with an asthma-exacerbation (24.3%) or musculoskeletal chest pain (20.5%).

**Table 1:** Baseline characteristics of study-cohort.

Variables	control (N = 263)	HF(N = 141)	p-value
**Gender**			
Male	163 (62.0%)	105 (74.5%)	0.01
Age* **(yrs)**	52.9 (±15.3)	61.4 (±11.1)	<0.001
**Ethnicity**			
Chinese	124 (47.1%)	71 (50.4%)	0.9
Malay	73 (27.8%)	39 (27.7%)	
Indian	46 (17.5%)	21 (14.9%)	
Other	20 (7.6%)	10 (7.1%)	
**Past history of smoking (n = 402)**	73 (27.8%)	45 (31.9%)	0.4
Body mass index* **(n = 158)**	27.2 (±6.6)	29.0 (±8.9)	0.2
**Medical history**			
Hypertension	128 (48.7%)	97 (68.8%)	<0.001
Diabetes	62 (23.6%)	76 (53.9%)	<0.001
Chronic Renal Impairment (n = 403)	14 (5.3%)	25 (17.7%)	<0.001
Obesity (n = 158)	32 (28.3%)	14 (31.1%)	0.4
Heart Failure		57 (40.4%)	<0.001
Anaemia (n = 375)	56 (23.9%)	75 (53.2%)	<0.001
Myocardial Infarction	28 (10.6%)	42 (29.8%)	<0.001
Cerebrovascular Accident	11 (4.2%)	13 (9.2%)	0.1
**Medication**			
beta-blocker (n = 398)	48(18.3%)	73(51.8%)	<0.001
ACE-I (n = 398)	23(8.7%)	48(34.0%)	<0.001
Statin (n = 400)	67(25.5%)	77(54.6%)	<0.001
Aspirin	45(17.1%)	71(50.4%)	<0.001
Diuretic (n = 401)	12(4.6%)	66(46.8%)	<0.001
**HFREF**		98 (77%)	
**HFPEF**		29 (23%)	
**NYHA class**			
unknown		44 (31.2%)	
Class 2		14 (9.9%)	
Class 3		46 (32.6%)	
Class 4		37 (26.2%)	
**Reduced (estimated) GFR (n = 241)**	17 (12%)	41 (40%)	<0.001
NTproBNP* **(pg/ml)**	882 (±3680)	7801 (±9100)	<0.001
**Diagnoses control group**			
Myocardial infarction	12 (4.6%)		
Unstable angina	16 (6.1%)		
Musculo-skeletal chest pain	54 (20.5%)		
Arrhythmia	8 (3.0%)		
COPD	15 (5.7%)		
Bronchitis	13 (4.9%)		
Asthma	64 (24.3%)		
Pneumonia-Bacterial	5 (1.9%)		
Pneumonia-Viral	1 (0.4%)		
Pulmonary Embolism	2 (0.8%)		
Influenza/viral syndrome	1 (0.4%)		
Others^†^	73 (27.8%)		

All numbers are presented as N (%) for categorical variables.

*Mean ± sd is shown for continuous variables.

Abbreviations: ACEI = ACE inhibitor; HFREF = heart failure with reduced ejection fraction; HFPEF = heart failure with preserved ejection fraction; GFR = glomerular filtration rate; COPD = chronic obstructive pulmonary disease

^†^others: diagnosis not specified: 42 patients, fluid overload due to end-stage renal failure: 2, acute myeloid leukaemia: 1, anaemia: 1, anaphylaxis: 3, biliary colic: 1, bronchiectasis: 1, cardiac tamponade: 1, chest infection: 1, cholecystitis/choledocholithiasis: 1, gastroenteritis: 2, hereditary haemorrhagic telangiectasia: 1, hyperventilation: 2, metastatic non-small cell lung carcinoma: 1, non-specific symptoms hyponatraemia: 1, oropharyngeal dysphagia: 1, palpitations: 3, panic attack: 1, parotiditis: 1, pleural effusion: 1, pneumothorax: 1, sepsis: 2, shortness of breath due to obesity: 1, symptomatic anaemia: 2, transient ischemic attack: 1, Upper respiratory tract infections: 3.

Baseline characteristics differed between patients with an adjudicated diagnosis of HF and controls (Table 1). Those with HF were older (mean age 61.4 year vs. 52.9 year) and more often male (74.5% vs. 62.0%) compared to the control group. Furthermore, the HF group carried higher percentages of hypertension, diabetes, chronic renal impairment, anemia, myocardial infarction and cerebrovascular accidents. Information on the ejection fraction was available for 95% of the newly diagnosed HF patients. 77% of HF cases had reduced left ventricle ejection fraction whereas 23% were HFPEF.

### Total extracellular vesicle-protein levels and Heart Failure

**Fig 1:**
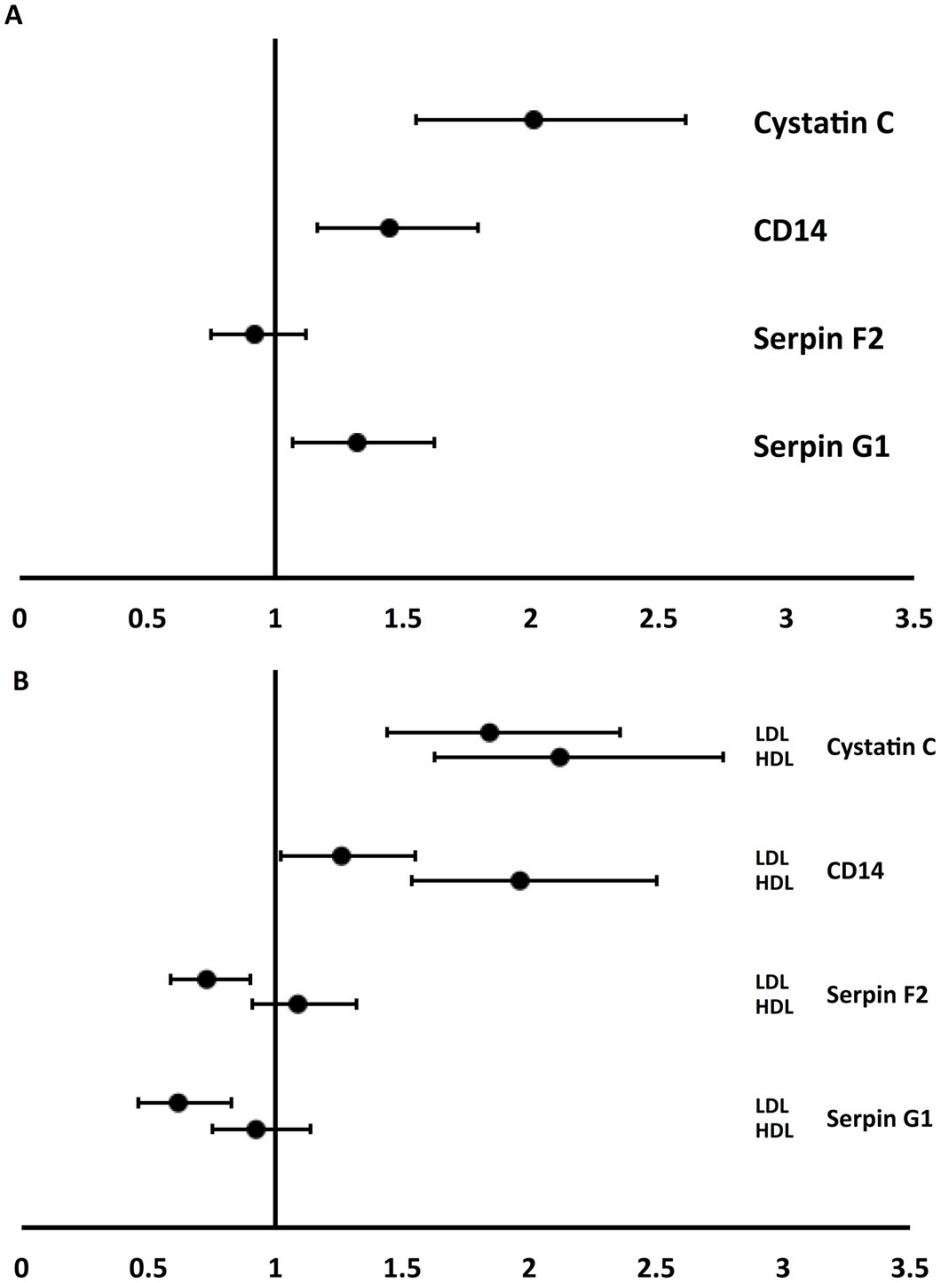
Relation between EV-markers and heart failure: forest plot of the uncorrected odds ratios with 95% confidence intervals. Reference groups were the patients without heart failure. 1-A: the uncorrected odds ratios with 95% confidence intervals for Total Extracellular vesicles (TEX); 1-B: the uncorrected odds ratios with 95% confidence intervals for LDL an HDL fractions EVs.

In our previous study, we demonstrated that CystatinC, SerpinF2, SerpinG1 and CD14-levels in the total extracellular vesicle (TEX)-population were related to vascular events [9]. TEX fraction, as described in method section, contains all extracellular vesicles in plasma. In the TEX population of the 4B-breathless cohort, levels of CystatinC (odds ratio (OR) 2.01; p-value <0.001), CD14 (OR 1.45, p = 0.001) and SerpinG1 (OR 1.32, p = 0.009) were significantly higher in heart failure-patients compared to controls (Fig 1A, median protein-levels in S1 Table), whereas SerpinF2 did not differ between these groups (OR 0.92, p = 0.44). All 4 proteins are present in extracellular vesicles as was shown by density gradient centrifugation (S4 Table).

To investigate if the association of EV protein levels with HF patients was independent of possible acute ischemic events, we limited the analysis to a subgroup of 26 heart-failure patients with no signs of myocardial ischemia and a control group of 217 patients with a low NT-proBNP. Similar associations between CystatinC (OR 2.01, p = 0.02), CD14 (OR 1.30, p = 0.2) and SerpinG1 (OR 1.43, p = 0.04) and heart failure were found.

In addition, the influence of other potential confounding variables on the relationship between EV-protein levels and HF was assessed using multivariable logistic regression analysis (see details on confounding variables in S2 Table). After adjustment for confounders, levels of TEX SerpinG1 (aOR 1.55, p = 0.004) and SerpinF2 (aOR 0.71, p = 0.021) remained statistically significantly related to heart failure (Table 2), whereas CD14 and CystatinC TEX-levels did not. CystatinC TEX protein level lost significance after correction for diuretics (S3 Table).

**Table 2:** Relation between EV-markers and heart failure, corrected for confounders (age, gender, hypertension, diabetes, chronic renal impairment, anemia, MI, Cerebrovascular accident (CVA), beta-blocker, ACE inhibitor, Statins, Aspirin, and diuretics).

	p-value	Odds ratio	95% C.I.for EXP(B)	
			Lower	Upper
Cystatin C				
TEX	0.97	1.01	0.70	1.44
LDL	0.27	1.18	0.88	1.58
HDL	0.28	1.20	0.86	1.69
CD14				
TEX	0.94	0.99	0.73	1.34
LDL	0.75	0.95	0.72	1.27
HDL	**0.01**	1.53	1.09	2.16
Serpin F2				
TEX	**0.02**	0.71	0.54	0.95
LDL	**0.01**	0.65	0.47	0.88
HDL	0.78	0.96	0.73	1.27
Serpin G1				
TEX	**0.00**	1.55	1.15	2.08
LDL	0.24	0.81	0.57	1.15
HDL	0.83	0.97	0.73	1.28

Reference groups were the patients without heart failure. Statistically significant differences are shown in bold.

### LDL- and HDL-associated extracellular vesicle-protein levels and heart failure

Having established that protein-levels in the total extracellular vesicle-population were related to heart failure, we examined protein-levels in subfractions of extracellular vesicles. Extracellular vesicles co-precipitating with LDL (LDL-EVs) exhibited higher levels of CystatinC (OR 1.84, p-value <0.001) and of CD14 (OR 1.26, p = 0.03) in heart failure-patients (Fig 1B). In contrast, SerpinF2 (OR 0.73, p = 0.007) and SerpinG1-levels (OR 0.62 p = 0.001) were significantly lower in cases compared to controls. All 4 proteins were present in extracellular vesicles as was shown by density gradient centrifugation (S4 Table). After adjustment for confounders, the relationship with HF remained for SerpinF2 LDL (aOR 0.65, p = 0.006) but not for CystatinC, CD14 and SerpinG1 in LDL-EVs (Table 2).

Extracellular vesicles co-precipitating with HDL (HDL-EVs) showed significantly increased levels of CystatinC (OR 2.12, p-value <0.001) and of CD14 (HDL 1.96, p<0.001) and the CD14 association persisted after correction for confounders (aOR 1.53, p = 0.014). However, there was no relation between HF and SerpinF2, CystatinC or SerpinG1 in HDL-EVs.

**Fig 2:**
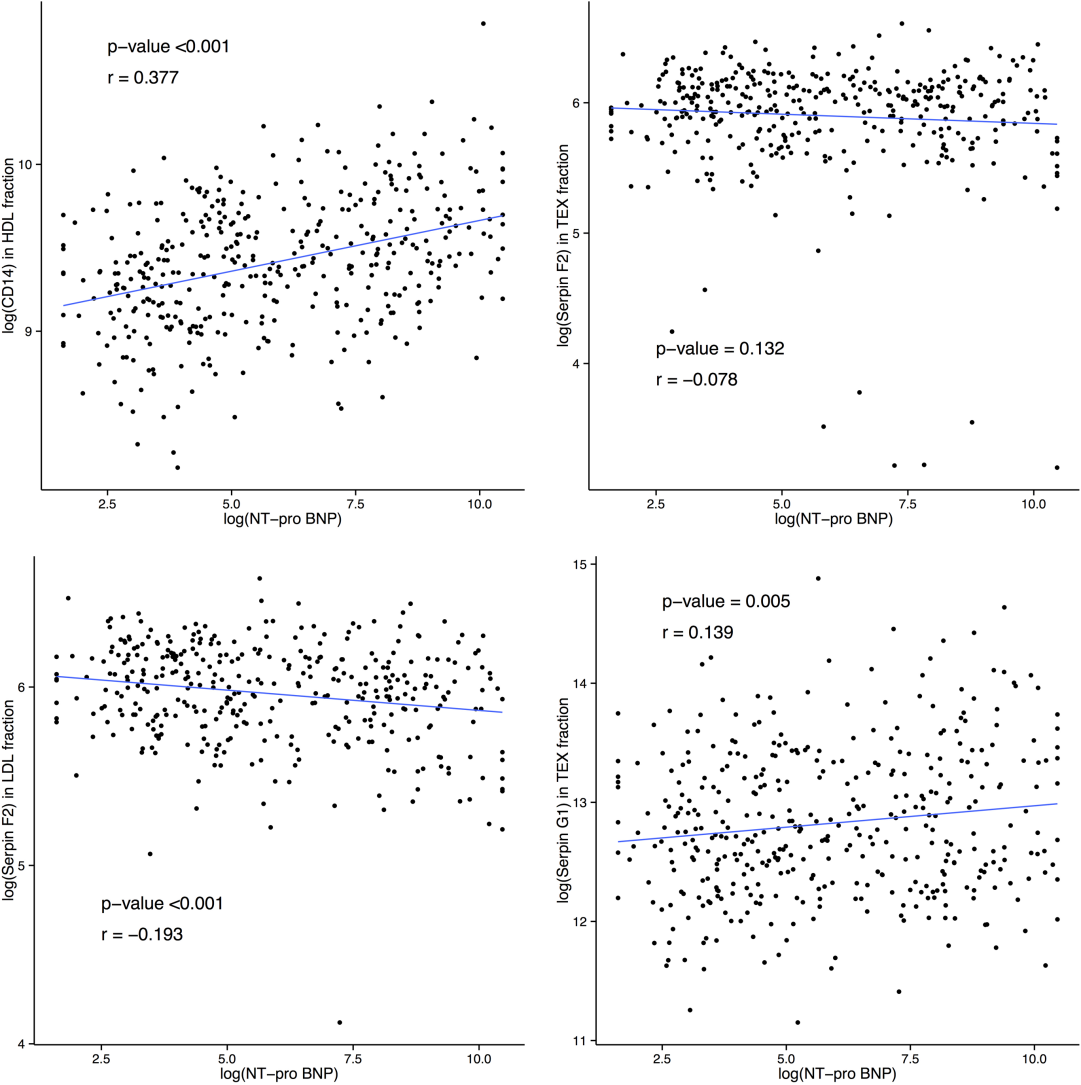
Correlation plots between N-terminal pro b-type natriuretic peptide (NT-proBNP) level and extracellular vesicle protein levels of CD14 in HDL fraction, Serpin F2 in TEX fraction, Serpin F2 in LDL fraction and Serpin G1 in TEX fraction. (r stands for correlation coefficient).

CD14 HDL, Serpin G1 TEX, Serpin F2 TEX and LDL remained statistically significant after correction and were correlated with the wall-stress HF marker NT-proBNP (Fig 2). CD14 level in HDL fraction (p<0.001, r = 0.377), SerpinF2 level in LDL fraction (p-value<0.001, r = -0.193) and SerpinG1 level in TEX fraction (p-value = 0.005, r = 0.139) were significantly correlated with NT-proBNP level. SerpinF2 level in TEX fraction showed no significant correlation to NT-proBNP level (p-value = 0.132, r = -0.078).

### Subgroup analysis: Extracellular vesicle protein levels in HFREF and HFPEF patients

HF-patients were further categorized into HFREF and HFPEF. After adjustment for confounders (age, gender, hypertension, diabetes, anemia and chronic renal impairment) however, the relationship between EV-proteins in the TEX fraction and HFPEF and HFREF disappeared (Table 3). However, The CystatinC and CD14 in HDL-fraction were significantly higher in HFREF patients (aOR 1.50, p = 0.034 and aOR 1.56, p = 0.038, respectively). SerpinF2 level in LDL fraction was significantly lower in HFREF patients (aOR 0.62, p = 0.029), as shown in Table 3. HFPEF patients had significantly higher SerpinF2 in HDL fraction but lower SerpinG1 in LDL fraction (aOR 1.76, p = 0.037 and aOR 0.12, p = 0.034, respectively).

**Table 3:** Relationship between EV-protein level and heart failure with reduced ejection fraction (HFREF) or heart failure with preserved ejection fraction (HFPEF) each compared to patients without heart failure (control).

Multivariable analysis	HFREF vs. control		HFPEF vs. control		HFREF vs. HFPEF		Multivariable analysis	HFREF vs. control			HFPEF vs. control			Multivariable analysis	HFREF vs. HFPEF		
	OR	p-value	OR	p-value	OR	p-value		OR	p-value	95%CI	OR	p-value	95%CI		OR	p-value	95%CI
Cystatin C							Cystatin C							Cystatin C			
TEX	1.46*	**0.01**	1.20*	0.32	0.95^5^	0.81	TEX	1.58	0.056	(0.99–2.52)	1.23	0.596	(0.58–2.60)	TEX	1.15	0.830	(0.32–4.12)
LDL	1.32^†^	0.06	1.40*	0.06	1.21^4^	0.46	LDL	1.38	0.117	(0.92–2.07)	0.99	0.985	(0.48–2.06)	LDL	1.29	0.748	(0.27–6.11)
HDL	1.40*	**0.02**	1.31*	0.09	1.31^4^	0.32	HDL	**1.50**	**0.034**	**(1.03–2.18)**	1.13	0.727	(0.57–2.22)	HDL	1.55	0.501	(0.43–5.58)
CD14							CD14							CD14			
TEX	1.03^‡^	0.83	1.21*	0.35	1.46^4^	0.15	TEX	1.03	0.912	(0.65–1.61)	1.46	0.257	(0.76–2.8)	TEX	0.39	0.088	(0.14–1.15)
LDL	0.82^‡^	0.21	1.58*	**0.01**	**2.24**^**6**^	**0.00**	LDL	0.90	0.641	(0.59–1.38)	1.41	0.245	(0.79–2.53)	LDL	0.29	0.062	(0.08–1.07)
HDL	**1.37**^**‡**^	**0.05**	2.29*	**0.00**	**1.87**^**4**^	**0.02**	HDL	**1.56**	**0.038**	**(1.03–2.38)**	1.94	0.096	(0.89–4.25)	HDL	0.65	0.412	(0.24–1.80)
Serpin F2							Serpin F2							Serpin F2			
TEX	0.82^‡^	0.19	1.04*	0.84	1.31^5^	0.24	TEX	0.70	0.104	(0.45–1.08)	1.07	0.834	(0.56–2.05)	TEX	0.70	0.453	(0.28–1.76)
LDL	**0.66**^**‡**^	**0.01**	1.04*	0.87	**1.64**^**5**^	**0.05**	LDL	**0.62**	**0.029**	**(0.4–0.95)**	1.10	0.762	(0.59–2.08)	LDL	0.60	0.223	(0.26–1.37)
HDL	0.86^‡^	0.31	1.70*	**0.01**	**2.18**^**5**^	**0.00**	HDL	0.85	0.431	(0.58–1.26)	**1.76**	**0.037**	**(1.04–2.99)**	HDL	**0.42**	**0.048**	**(0.18–0.99)**
Serpin G1							Serpin G1							Serpin G1			
TEX	**1.37**^**‡**^	**0.04**	1.31*	0.21	0.98^5^	0.94	TEX	1.40	0.083	(0.96–2.03)	1.04	0.925	(0.45–2.43)	TEX	1.96	0.414	(0.39–9.91)
LDL	0.94^‡^	0.67	0.24*	**0.01**	0.51^4^	0.19	LDL	0.84	0.506	(0.50–1.40)	**0.12**	**0.034**	**(0.02–0.85)**	LDL	2.66	0.410	(0.26–27.18)
HDL	0.96*	0.77	0.94*	0.77	1.00^5^	0.98	HDL	1.23	0.298	(0.83–1.82)	0.77	0.535	(0.35–1.74)	HDL	1.76	0.359	(0.52–5.94)

Analysis of the EV-subfractions elucidated a difference between HFREF and HFPEF. SerpinF2-level in HDL fraction was significantly lower in HFREF-patients (Table 4).

**Table 4:** Relationship between EV-protein level and heart failure with reduced ejection fraction (HFREF) compared to heart failure with preserved ejection fraction (HFPEF).

Multivariable analysis	HFREF vs. HFPEF		
	OR	p-value	95%CI
Cystatin C			
TEX	1.15	0.830	(0.32–4.12)
LDL	1.29	0.748	(0.27–6.11)
HDL	1.55	0.501	(0.43–5.58)
CD14			
TEX	0.39	0.088	(0.14–1.15)
LDL	0.29	0.062	(0.08–1.07)
HDL	0.65	0.412	(0.24–1.80)
Serpin F2			
TEX	0.70	0.453	(0.28–1.76)
LDL	0.60	0.223	(0.26–1.37)
HDL	**0.42**	**0.048**	**(0.18–0.99)**
Serpin G1			
TEX	1.96	0.414	(0.39–9.91)
LDL	2.66	0.410	(0.26–27.18)
HDL	1.76	0.359	(0.52–5.94)

Adjustment for confounders (age, gender, hypertension, diabetes, anemia and chronic renal impairment). OR = odds ratio.

### Subgroup analysis: Extracellular vesicle protein levels in HF patients with or without MI history

Within the HF group, we further analyzed the EV protein levels between HF patients with or without history of MI. SerpinG1 level in TEX fraction was significantly different between HF patients with or without MI (p = 0.020 in multivariable analysis, S7 Table).

Because a significant interaction was found (p_interaction_ = 0.027) between history of MI and CD14 levels in the HDL fraction for the diagnosis of HF in the logistic regression model, the analysis was further stratified (interactions with all other proteins were not significant). For patients without a history of MI there was no significant relation between CD14 levels in the HDL fraction (aOR 1.3, p-value = 0.365) but for patients with a history of MI it was related to HF (aOR 3.1, p-value = 0.041).

## Discussion

Systemic vascular disease with an underlying pro-inflammatory status is thought to contribute to the development and progression of HF [18, 22, 30]. We investigated if extracellular vesicle markers for systemic vascular disease differed between HF and non-HF patients. In this observational study in an emergency department cohort of dyspnea patients (4B), we showed for the first time that these vascular disease EV markers are indeed different in HF patients, which might point to a common underlying pathophysiology of HF.

The 4B dyspnea cohort has a comparable percentage of HF diagnoses to other dyspnea cohorts [31–33]. Furthermore, the high prevalence of hypertension and diabetes among HF patients is in line with other HF cohorts [31–33]. Although the percentage of diabetes seems higher in the HF patients of the 4B cohort, a comparable diabetes-prevalence has been described in a different but contemporaneous Singaporean HF cohort [34] and might be linked to differences in ethnicity as also observed in coronary artery disease [35].

Numbers of plasma EVs with endothelial cell markers were previously found to be related to the functional HF NYHA class [7]. We now describe that levels of EV proteins associated with systemic vascular events, are also associated with the presence of HF.

### Cystatin C

CystatinC, a marker of renal function [10], is higher in all plasma fractions in HF in univariable analysis but loses significance after correction for comorbidities. After correction for chronic renal impairment or reduced GFR elevated CystatinC EV-levels are still independently associated with HF, suggesting a kidney-independent relation of CystatinC with HF. This relationship, however, loses significance after correction for diuretic use. Diuretics have shown to modify plasma CystatinC levels [36]. Our data show that diuretic use is also associated with EV CystatinC levels.

### CD14

CD14 is a co-receptor of Toll-like receptor 4, a key receptor of the innate immune system [37]. In our study CD14 was found to be independently associated with HF, confirming the important role of the innate immune system in HF [38]. Previously it has been shown that the CD14-260C/T polymorphism, which leads to an increase in CD14-receptor density [39] is associated with HF [40]. CD14-positive monocytes have also shown to be increased in HF [41, 42], which might be directly related to our finding of increased EV CD14 in HF. While soluble CD14 levels have been reported to be higher in chronic HF patients than in controls [43], the CD14 measured in this study is present in vesicles ^7^ and is therefore very likely to be the anchored membrane CD14 protein This is in agreement with the association of the increase of CD14 positive monocytes with echo parameters for diastolic function [41, 42]. Furthermore, monocyte derived EVs, marked by CD14, are pro-coagulant [15]. It is known that HF patients are more prone to thrombotic events such as venous thromboembolism and pulmonary embolism [44]. Diminished blood flow in the dilated atria and ventricles might be (partially) responsible for the development of thrombi but this could also be due to a more activated coagulation system and enhanced platelet function. Although thrombus formation is an important and well-known process involved in the occurrence of vascular events [45], it’s role in HF is largely unknown.

### Serpin F2

Plasma SerpinF2 (alpha-2-plasmin inhibitor) reduces fibrinolysis [14] and thereby the degradation of thrombi. In a previous study, higher SerpinF2-levels in EVs were related to an increased risk for myocardial infarction and vascular mortality [9]. On the other hand, Matsuno et al. found that a lack of SerpinF2 in mice promoted pulmonary heart failure via release of VEGF after acute myocardial infarction [46]. SerpinF2-levels have not been associated with HF in humans before. In our study, EV SerpinF2 in the LDL fraction was lower in HF-patients. Plasma SerpinF2 is mainly produced in the liver and kidney and was found to be reduced in patients with nephrotic syndrome [47]. The lower EV-SerpinF2 levels in our study could be caused by a reduced production related to a compromised kidney function in HF-patients. Although a reduction in SerpinF2 might lead to an increase in fibrinolysis, thereby possibly protecting against the development of vascular mortality and HF, the VEGF- related effects of a lower SerpinF2 might prevail in the association between SerpinF2 and HF. It remains unclear, however, whether a possible pro-thrombotic state and/or systemic inflammation in HFPEF are secondary to HF, or whether these are involved in cause or progression of HFPEF. Validated HFPEF animal models or early antithrombotic interventions are needed to answer this question.

### Serpin G1

SerpinG1, or C1-inhibitor, is an acute phase protein and regulates complement activation. EV-Serpin G1 was previously found to be positively correlated with hsCRP [9, 48]. We found that EV-SerpinG1 in the TEX fraction was associated with higher odds of HF, which points to an increased inflammatory state in these patients. Serpin G1 TEX levels in the HF patient group differ between HF patients with and without MI which is in accordance with the association of SerpinG1 with ischemia [49, 50].

The association of the 4 EV protein markers for systemic vascular events with HF points to possible common mechanisms. All 4 EV proteins are associated with systemic inflammation, which is characteristic of risk factors (like diabetes and hypertension) for both vascular events and HF. The currently investigated EV-proteins have been associated with vascular events; and in turn, vascular events like myocardial infarction can lead to acute HF. However, patients with signs and symptoms of a myocardial infarction were classified as such and not as acute HF-patients. Furthermore, subgroup analysis of a relative homogenous subgroup of HF patients (low hsTnT and no ECG changes) with a homogenous non-HF group (low NT-proBNP) showed that differences between HF and non-HF patients remained, suggesting that the association of EV proteins with HF is probably independent of underlying ischemic events.

### Plasma EV sub-fractions

We found more marked differences between HF and non-HF patients in SerpinF2- and SerpinG1 in the LDL-EV-fractions. Plasma EV sub-fractions are unexplored with unknown biological and pathophysiological functions, providing a new arena of research. LDL- and HDL-plasma fractions each have a specific subset of EVs [2], which could reflect different origins and might point to different functions.

### NT-proBNP

Since NT-proBNP is released when the ventricle wall encounters an overload of stress [51], the significant correlations between NT-proBNP level and CD14 level in HDL fraction, SerpinF2 level in LDL fraction as well as SerpinG1 level in the TEX fraction suggest that these EV proteins are related to wall stress. In contrast, SerpinF2 TEX did not correlate with NT-proBNP, which possibly indicates a role in a non-BNP-related mechanism.

### Strengths and limitations

The present study describes the association between EV-protein levels and HF in an undifferentiated breathless cohort with careful adjudication of the cause of breathlessness. Another strength of our study is that the control group consisted of non-healthy patients, rendering it more likely that differences in EV-protein levels were due specifically to pathophysiological processes associated with HF and not due to less specific effects of illness.

The plasma protein levels of CD14, SerpinF2 and SerpinG1 only partly differed between HF and controls (S5 Table). There was no significant correlation between plasma protein levels and the EV sub-fraction levels (S6 Table) showing that EV protein levels contain different information than plasma protein levels. Although reproducibility of the isolation of EV fractions is good (coefficient of variation (CV) <15%, data not shown), we do not know the percentage of EVs in each fraction compared to the total amount of plasma EVs. This seems to be a major limitation of the currently available EV isolation techniques that may lead to investigation of only minor fractions of total EVs of the blood plasma [52].

Although we corrected for the most important confounders, residual confounding cannot be excluded. Therefore, other cohort studies are needed to confirm our results. The mechanisms behind the differential correlation between the protein levels in different fractions and HF remain unknown. The causality of EVs in endothelial dysfunction leading to HF has not been studied yet. Further studies are needed to investigate the origin of the EVs.

## Conclusion

In summary, our study shows that EV markers for systemic vascular events (CD14, SerpinF2 and SerpinG1) are associated with HF and suggests possible common underlying pathophysiological mechanisms as systemic inflammation but perhaps also thrombosis for both HF and vascular events.

## Supporting Information

S1 Fig Flowchart of N = 617 Patients Reaching the Emergency Department with Complaint of Breathlessness in the Observational, Cross-Sectional Study of 4B-Cohort. (PDF)

S2 Fig Extracellular Vesicles in LDL and HDL Fraction Under Electron Microscopy (Bar stands for 200nm). (PDF)

S3 Fig Western Blot Showing the Distribution of CD9 Level in TEX, LDL and HDL Fractions with Density Gradient Experiment. (PDF)

S1 File Supplemental Methods. (PDF)

S1 Table Median Levels of EV-Proteins (in ng/mL) in Controls and in Patients with Heart Failure (HF). (PDF)

S2 Table Effect of Potential Confounders on EV-Protein-Levels. (PDF)

S3 Table Relation between EV-Markers and Heart Failure, Corrected for Confounders (Age, Gender, Hypertension, Diabetes, Chronic Renal Impairment Anemia, MI, CVA, Beta-Blocker, ACE Inhibitor, Statins and Aspirin but Not for Diuretics). (PDF)

S4 Table The Distribution of CD14, SerpinG1, SerpinF2 And CystatinC in TEX (S4-1), LDL (S4-2) and HDL (S4-3) Fractions with Density Gradient Experiment. (PDF)

S5 Table Comparing CD14, SerpinF2, and SerpinG1 Levels in Plasma, TEX, LDL- and HDL Fractions Respectively between HF and Non-HF Groups. (PDF)

S6 Table Correlation of Protein Levels (CD14, SerpinF2 And SerpinG1) Comparing Plasma Protein Levels against in TEX, LDL- and HDL- EV Fractions. (PDF)

S7 Table The Comparison of EV Protein Levels in HF Patients With and Without MI History. (PDF)
