# Supplementary material for: Extracellular Vesicle Proteins Associated with Systemic Vascular Events Correlate with Heart Failure: An Observational Study in a Dyspnoea Cohort
Source: PLoS One. 2016 Jan 28;11(1):e0148073. doi: 10.1371/journal.pone.0148073 (PMC4731211; doi:10.1371/journal.pone.0148073)
Supplement: S1 Fig — (PDF) [file pone.0148073.s001.pdf]

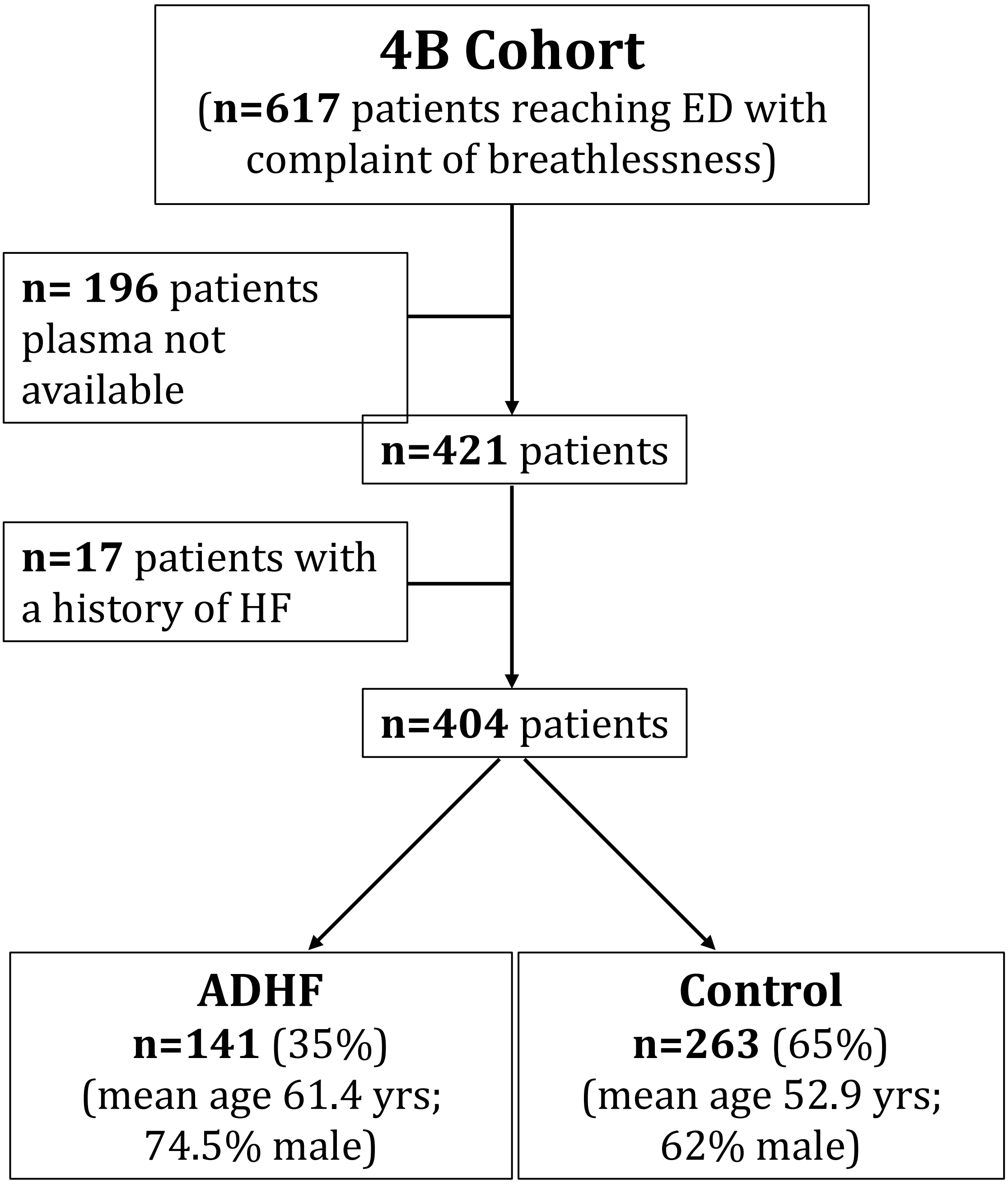

S1 Fig. Flowchart of n=617 patients reaching the emergency department with complaint of breathlessness in the observational, cross-sectional study of 4B-cohort. ED: emergency department; HF: heart failure; ADHF: acute decompensated heart failure.
