# Supplementary material for: Extracellular Vesicle Proteins Associated with Systemic Vascular Events Correlate with Heart Failure: An Observational Study in a Dyspnoea Cohort
Source: PLoS One. 2016 Jan 28;11(1):e0148073. doi: 10.1371/journal.pone.0148073 (PMC4731211; doi:10.1371/journal.pone.0148073)
Supplement: S2 Fig — (PDF) [file pone.0148073.s002.pdf]

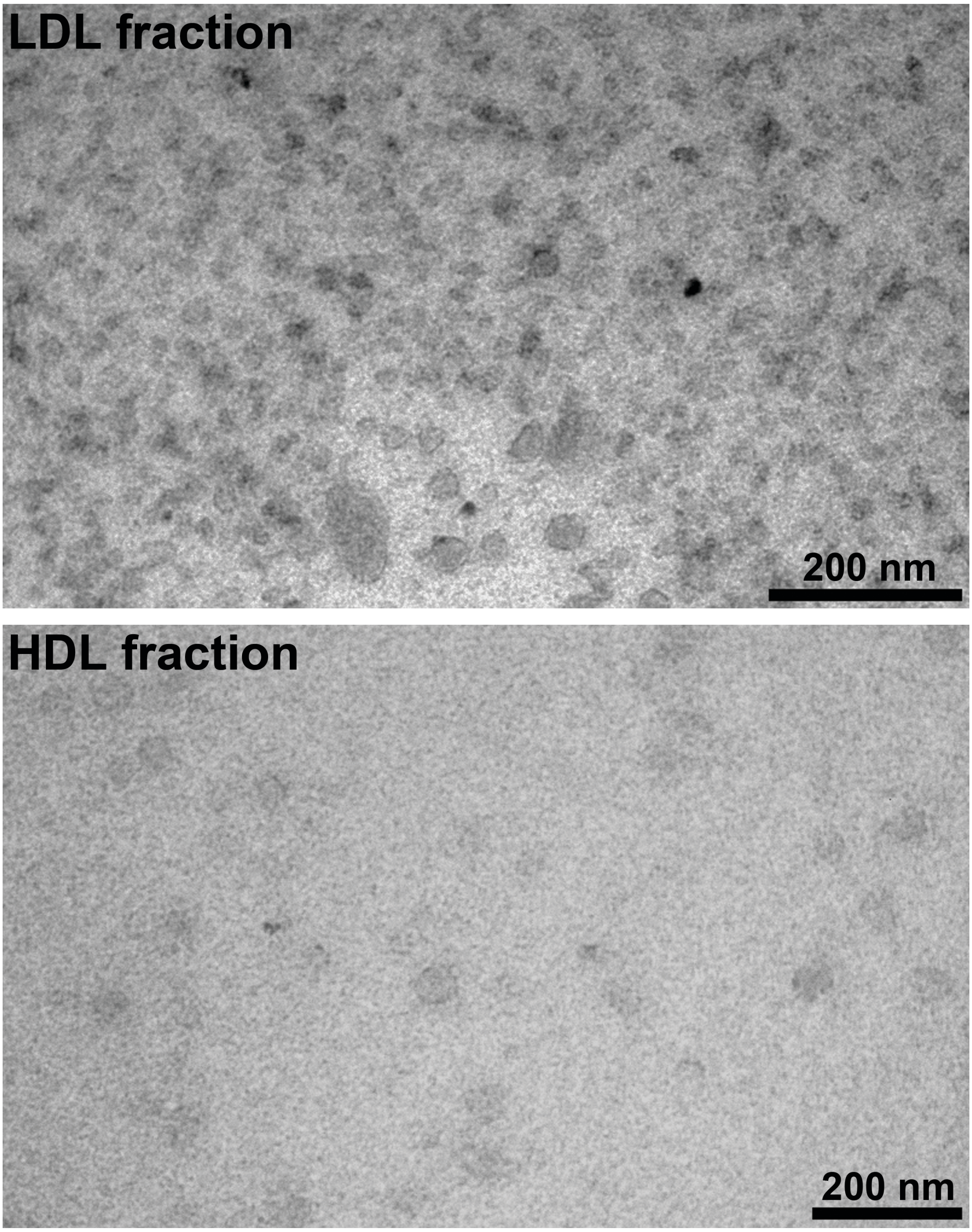

S2 Fig. Extracellular Vesicles in LDL and HDL Fraction Under Electron Microscopy. The electron microscopy experiments were conducted as described in S1_File. Bar stands for 200 nm.
