# Supplementary material for: Extracellular Vesicle Proteins Associated with Systemic Vascular Events Correlate with Heart Failure: An Observational Study in a Dyspnoea Cohort
Source: PLoS One. 2016 Jan 28;11(1):e0148073. doi: 10.1371/journal.pone.0148073 (PMC4731211; doi:10.1371/journal.pone.0148073)
Supplement: S3 Fig — (PDF) [file pone.0148073.s003.pdf]

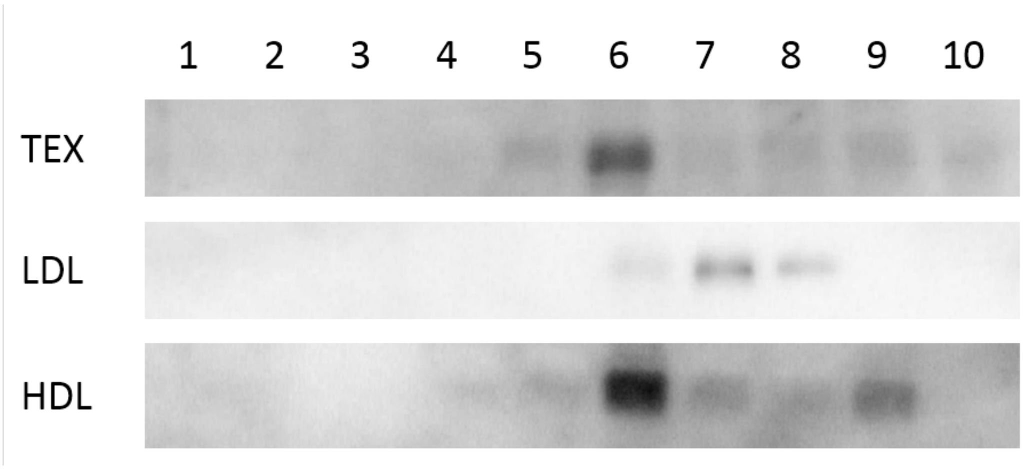

S4 Fig. Western Blot Showing the Distribution of CD9 Level in TEX, LDL and HDL Fractions with Density Gradient Experiment. Numbers on top stands for the sub-fractions after density gradient experiments. The sub-fractions were the same ones as in S4 Table. The density of each subfraction is listed in S4 Table. The Western Blot method refers to S1_File.
