# Supplementary material for: Extracellular Vesicle Proteins Associated with Systemic Vascular Events Correlate with Heart Failure: An Observational Study in a Dyspnoea Cohort
Source: PLoS One. 2016 Jan 28;11(1):e0148073. doi: 10.1371/journal.pone.0148073 (PMC4731211; doi:10.1371/journal.pone.0148073)
Supplement: S1 File — (PDF) [file pone.0148073.s004.pdf]

Supplemental Method:

Density gradient experiment

Five buffers with discontinous density was prepared with OptiPrep™ Density Gradient Medium (OptiPrep, Axis-Shield# 1114542), 10x PBS (Ambion™# AM9624) and ddH_2O. The end composition of these 5 buffers contained 5%, 10%, 20%, 30% and 40% of OptiPrep in 1x PBS respectively. The more OptiPrep, the higher the density. Two mL of each buffer was carefully added and overlaid sequentially (starting from the highest density) in to the ultracentrifuge tubes (Beckman Coulter# 344059). Pellets of TEX, LDL and HDL fraction isolated from 12mL plasma according to the methods above were resuspended in 500μL 1x PBS and also added in to the ultracentrifuge tubes respectively. After spinning at 200,000g for 18h at 4ºC (Beckman Coulter# Optima XL-90 Ultracentrifuge, #SW 41 Ti Rotor), each solution in the ultracentrifuge tubes was collected from top to bottom sequentially in 10 Eppendorf tubes (1mL/sub-fraction, the last one with excess leftover). Sub-fractions were vortexed thoroughly; 900μL of each was added in new ultracentrifuge tube respectively together with 7mL 1xPBS (containing 0.1% BSA) and then spun at 200,000g for 1h at 4ºC; the rest 100μL of each sub-fraction was used for density calculation by mass dividing volume. The pellets were dissolved in 200μL Roche lysis buffer; subsequently, the protein quantity was measured with the methods described in the main manuscript.

Plasma protein level analysis

The protein levels of CD14, SerpinF2 and SerpinG1 were directly measured in plasma with the methods described in the main text; in total, plasma samples from 370 subjects (126 HF patients and 244 Non-HF patients) were measured in this step based on the availability of the plasma. The difference of plasma CD14, SerpinF2 and SerpinG1 levels between HF and Non-HF groups was calculated with Mann-Whitney U test. The correlation of the protein levels in EV fractions (TEX, LDL and

HDL) and in plasma was analysis with Pearson bivariate correlation analysis. Both analyses were processed with SPSS® (IBM®, Version 22.0.0.0).

Electron Microscopy

Both the LDL-EV fraction (pellet after isolation was resuspended in 1x PBS) and its sub-fractions from density gradient centrifugation (100µL leftover of each sub-fraction) were processed at room temperature (20ºC) for electron microscopy (EM). The samples were diluted 1:5 in 1x PBS prior to fixation with 2.0% glutaraldehyde (Sigma Aldrich #G5882). After fixation, a 75-mesh grid (Agar scientific#G2075C) was laid on a drop of sample for 10 min; then the grid was rinsed 10 times with MiliQ H2O (1 min per rinse). For staining, the grid was firstly laid on a drop of uranyl acetate (pH 7.0, SPI-CHEM# 2624) for 10 min. After rinsing, with Milli-Q H2O and methylcellulose uranyl (pH 4.0), the grid was incubated for 10 min on a drop of methylcellulose uranyl (pH 4.0, Sigma Aldrich#M-6385). The samples were analyzed with an FEI Tecnai™ T12 electron microscope.

Western Blot

Western blot was done as described before[1]. The antibodies used in western blot for CD9: CD9 Antibody (C-4) (santa cruz biotechnology#SC13118, primary antibody), Polyclonal Goat Anti-Mouse Immunoglobulins (Dako# P0447, secondary antibody). For visualization, the ECL kit HRP (Substrate), Immobilon Western Chemiluminescent HRP substrate (Milipore #WBKLS0500), was used. The sub-fractions after density gradient experiment were lysed with 0.1% Sodium dodecyl sulfate (SDS) for Western Blot. Only for the sub-fraction of HDL, 0.5% SDS was used for lysing.

Reference

1. Kanhai D. Adiposity, adipocytokines & microvesicles in the etiology of vascular disease. 2013.
