# Supplementary material for: Extracellular Vesicle Proteins Associated with Systemic Vascular Events Correlate with Heart Failure: An Observational Study in a Dyspnoea Cohort
Source: PLoS One. 2016 Jan 28;11(1):e0148073. doi: 10.1371/journal.pone.0148073 (PMC4731211; doi:10.1371/journal.pone.0148073)
Supplement: S1 Table — (PDF) [file pone.0148073.s005.pdf]

S1 Table. Median levels of EV-proteins (in ng/ml) in controls and in patients with heart failure (HF).

		Control<br>Median (IQR)	HF<br>Median (IQR)
Cystatin C	TEX	558.9 (404.5-752.8)	840.1 (520.2-1360.5)
	LDL	177.3 (95.4-268.2)	252.4 (167.1-383.8)
	HDL	37.5 (26.8-50.8)	60.8 (42.2-97.7)
CD14	TEX	163.7 (126.4-209.9)	185.7 (147.3-250.6)
	LDL	88.1 (68.4-105.2)	90.9 (73.4-128.5)
	HDL	11.3 (8.7-14.6)	14.3 (11.8-18.7)
Serpin F2	TEX	953.2 (567.6-1381.1)	943 (535.8-1291.8)
	LDL	1065 (641.1-1511.3)	885.7 (487.9-1279.6)
	HDL	73.8 (38.1-123.7)	80.5 (46.3-133.8)
Serpin G1	TEX	328.4 (222.6-542.2)	416.4 (262.1-669)
	LDL	1122.9 (749.7-1754)	754.3 (536.2-1230.4)
	HDL	192.7 (139.7-268.7)	180.6 (127.2-262.2)

IQR= interquartile range
