# Supplementary material for: Extracellular Vesicle Proteins Associated with Systemic Vascular Events Correlate with Heart Failure: An Observational Study in a Dyspnoea Cohort
Source: PLoS One. 2016 Jan 28;11(1):e0148073. doi: 10.1371/journal.pone.0148073 (PMC4731211; doi:10.1371/journal.pone.0148073)
Supplement: S2 Table — (PDF) [file pone.0148073.s006.pdf]

S2 Table. Effect of potential confounders on EV-protein-levels.

		Age	Gender	HT	Diabetes	CRI	Anemia	MI	CVA	β-blocker	ACEI	Statins	Aspirin	Diuretics
	TEX	0.04	0.01	0.23	0.03	0.57	0.01	0.52	0.30	0.86	0.01	0.04	0.02	0.00
Cystatin C	LDL	0.04	0.02	0.25	0.04	0.75	0.01	0.51	0.30	0.79	0.01	0.05	0.02	0.00
	HDL	0.06	0.01	0.26	0.03	0.93	0.01	0.46	0.27	0.84	0.02	0.05	0.02	0.00
	TEX	0.04	0.02	0.23	0.03	0.50	0.01	0.53	0.30	0.85	0.01	0.04	0.02	0.00
CD14	LDL	0.04	0.01	0.23	0.02	0.48	0.01	0.55	0.31	0.88	0.01	0.04	0.02	0.00
	HDL	0.08	0.01	0.28	0.05	0.78	0.01	0.41	0.20	0.75	0.01	0.06	0.03	0.00
	TEX	0.03	0.03	0.31	0.04	0.81	0.00	0.53	0.32	0.90	0.02	0.04	0.01	0.00
Serpin F2	LDL	0.06	0.02	0.47	0.04	0.85	0.00	0.63	0.36	0.96	0.01	0.04	0.01	0.00
	HDL	0.04	0.01	0.25	0.02	0.53	0.01	0.53	0.30	0.88	0.01	0.04	0.02	0.00
	TEX	0.05	0.01	0.22	0.02	0.56	0.02	0.30	0.32	0.95	0.03	0.04	0.02	0.00
Serpin G1	LDL	0.06	0.01	0.31	0.03	0.58	0.01	0.53	0.29	0.67	0.01	0.03	0.03	0.00
	HDL	0.04	0.02	0.24	0.02	0.48	0.01	0.53	0.31	0.84	0.02	0.04	0.02	0.00

P-values were calculated by 2-way ANCOVA, corrected for heart failure.

HT=hypertension; CRI=chronic renal impairment; MI=myocardial infarction;

CVA=cerebrovascular accident; ACEI=ACE inhibitor.
