# Supplementary material for: Extracellular Vesicle Proteins Associated with Systemic Vascular Events Correlate with Heart Failure: An Observational Study in a Dyspnoea Cohort
Source: PLoS One. 2016 Jan 28;11(1):e0148073. doi: 10.1371/journal.pone.0148073 (PMC4731211; doi:10.1371/journal.pone.0148073)
Supplement: S3 Table — (PDF) [file pone.0148073.s007.pdf]

S3 Table. Relation between EV-markers and heart failure, corrected for confounders (age, gender, hypertension, diabetes, chronic renal impairment anemia, MI, CVA, beta-blocker, ACE inhibitor, Statins and Aspirin but not for diuretics).

		p-value	Odds ratio	95% C.I.for EXP(B)	
				Lower	Upper
Cystatin C	TEX	**0.043**	1.377	1.010	1.878
	LDL	**0.045**	1.320	1.006	1.733
	HDL	**0.014**	1.459	1.079	1.973
CD14	TEX	0.355	1.127	0.875	1.451
	LDL	0.861	1.022	0.801	1.305
	HDL	**0.002**	1.535	1.168	2.018
Serpin F2	TEX	0.128	0.822	0.639	1.058
	LDL	**0.008**	0.709	0.551	0.914
	HDL	0.994	1.001	0.795	1.260
Serpin G1	TEX	**0.004**	1.456	1.125	1.885
	LDL	0.174	0.813	0.604	1.095
	HDL	0.742	0.961	0.757	1.219

Reference groups were the patients without heart failure. Statistically significant differences are shown in bold.
