# Supplementary material for: Extracellular Vesicle Proteins Associated with Systemic Vascular Events Correlate with Heart Failure: An Observational Study in a Dyspnoea Cohort
Source: PLoS One. 2016 Jan 28;11(1):e0148073. doi: 10.1371/journal.pone.0148073 (PMC4731211; doi:10.1371/journal.pone.0148073)
Supplement: S4 Table — (PDF) [file pone.0148073.s008.pdf]

S4 Table. The distribution of CD14, SerpinG1, SerpinF2 and CystatinC in TEX (4-1), VLDL (4-2) and HDL (4-3) fractions with density gradient experiment.

S4 Table 4-1. The distribution of CD14, SerpinG1, SerpinF2 and CystatinC in TEX fraction

Sub-fraction	CD14	SerpinG1	SerpinF2	CystatinC	Density (g/ml)
TEX-1	0.00*	0.00	0.00	0.00	0.90
TEX-2	0.00	0.00	0.00	0.00	0.94
TEX-3	0.00	0.00	0.00	0.00	0.98
TEX-4	105.15	0.00	214.41	0.00	1.01
TEX-5	142.52	12332.43	5816.29	897.97	1.05
TEX-6	62.53	15321.08	6100.78	352.06	1.09
TEX-7	0.00	1802.16	1209.81	427.60	1.14
TEX-8	0.00	0.00	360.40	0.00	1.18
TEX-9	0.00	0.00	175.10	0.00	1.24
TEX-10	0.00	0.00	118.75	0.00	1.40

S4 Table 4-2. The distribution of CD14, SerpinG1, SerpinF2 and CystatinC in LDL fraction

Sub-fraction	CD14	SerpinG1	SerpinF2	CystatinC	Density (g/ml)
LDL-1	0.00	1324.24	117.16	322.77	0.90
LDL-2	0.00	0.00	170.36	0.00	0.93
LDL-3	48.84	0.00	72.88	0.00	0.96
LDL-4	0.00	0.00	0.00	0.00	0.98
LDL-5	0.00	0.00	68.82	0.00	1.00
LDL-6	56.41	2167.79	159.29	0.00	1.02
LDL-7	56.87	43675.69	446.41	0.00	1.05
LDL-8	0.00	34964.50	536.44	253.44	1.08
LDL-9	0.00	0.00	0.00	0.00	1.17
LDL-10	0.00	0.00	64.75	0.00	1.35

S4 Table 4-3. The distribution of CD14, SerpinG1, SerpinF2 and CystatinC in HDL fraction

Sub-fraction	CD14	SerpinG1	SerpinF2	CystatinC	Density (g/ml)
HDL-1	0.00	0.00	0.00	0.00	0.90
HDL-2	0.00	0.00	0.00	0.00	0.93
HDL-3	0.00	0.00	0.00	0.00	0.97
HDL-4	0.00	0.00	0.00	0.00	1.00
HDL-5	0.00	1977.26	313.29	0.00	1.04
HDL-6	247.02	14729.33	5753.63	1112.32	1.08
HDL-7	126.88	18708.66	7652.38	1420.24	1.12
HDL-8	102.98	10844.09	13190.30	1102.92	1.17
HDL-9	0.00	11380.27	4999.12	0.00	1.29
HDL-10	52.21	2044.45	5605.83	501.27	1.39

*, 0.00 stands for the concentration was below Lower Limit of Quantification. The unit of the protein concentration is μg/mL.
