# Supplementary material for: Extracellular Vesicle Proteins Associated with Systemic Vascular Events Correlate with Heart Failure: An Observational Study in a Dyspnoea Cohort
Source: PLoS One. 2016 Jan 28;11(1):e0148073. doi: 10.1371/journal.pone.0148073 (PMC4731211; doi:10.1371/journal.pone.0148073)
Supplement: S5 Table — (PDF) [file pone.0148073.s009.pdf]

S5 Table. Comparing CD14, SerpinF2, and SerpinG1 levels in plasma, TEX, LDL- and HDL fractions respectively between HF and Non-HF groups. Table contains the different p-value's after Mann Whitney test between HF and Non-HF

	Plasma	TEX	HDL	LDL
CD14	0.14	0.00	0.00	0.04
SerpinF2	0.00	0.13	0.96	0.00
SerpinG1	0.00	0.02	0.17	0.00
