# Supplementary material for: Extracellular Vesicle Proteins Associated with Systemic Vascular Events Correlate with Heart Failure: An Observational Study in a Dyspnoea Cohort
Source: PLoS One. 2016 Jan 28;11(1):e0148073. doi: 10.1371/journal.pone.0148073 (PMC4731211; doi:10.1371/journal.pone.0148073)
Supplement: S6 Table — (PDF) [file pone.0148073.s010.pdf]

S6 Table. Correlation of protein levels (CD14, SerpinF2 and SerpinG1) comparing plasma protein levels against in TEX, LDL- and HDL- EV fractions.

		correlation coefficient	P-value
CD14	HDL	0.010	0.834
	LDL	-0.018	0.721
	TEX	-0.026	0.600
SerpinF2	HDL	-0.075	0.152
	LDL	-0.026	0.624
	TEX	-0.015	0.770
SerpinG1	HDL	0.030	0.553
	LDL	-0.033	0.502
	TEX	-0.012	0.805
