# Supplementary material for: Extracellular Vesicle Proteins Associated with Systemic Vascular Events Correlate with Heart Failure: An Observational Study in a Dyspnoea Cohort
Source: PLoS One. 2016 Jan 28;11(1):e0148073. doi: 10.1371/journal.pone.0148073 (PMC4731211; doi:10.1371/journal.pone.0148073)
Supplement: S7 Table — (PDF) [file pone.0148073.s011.pdf]

S7 Table. The comparision of EV protein levels in HF patients with and without MI history

		p-value	Mean Difference	Std. Error Difference	95%CI
Cystatin C	TEX	0.349	-123556.02	131368.30	(-383294.5, 136182.4)
	LDL	0.604	-19853.40	38216.09	(-95413.4, 55706.6)
	HDL	0.375	-6153.67	6909.23	(-19814.4, 7507.1)
CD14	TEX	0.426	12825.51	16070.13	(-18948, 44599)
	LDL	0.668	3277.26	7632.39	(-11813.3, 18367.9)
	HDL	0.621	-560.01	1131.45	(-2797.1, 1677.1)
Serpin F2	TEX	0.790	-32375.68	121424.27	(-272453, 207701.7)
	LDL	0.970	-4419.06	115560.84	(-232903.4, 224065.3)
	HDL	0.844	2506.49	12706.75	(-22617, 27630)
Serpin G1	**TEX**	**0.011**	**157899.12**	**61248.21**	**(36435.8, 279362.4)**
	LDL	0.846	33738.29	172968.73	(-308251.6, 375728.2)
	HDL	0.534	13803.71	22122.82	(-29937, 57544.5)
